# Matrin 3 in neuromuscular disease: physiology and pathophysiology

**DOI:** 10.1172/jci.insight.143948

**Published:** 2021-01-11

**Authors:** Ahmed M. Malik, Sami J. Barmada

**Affiliations:** 1 Medical Scientist Training Program,; 2 Neuroscience Graduate Program, and; 3 Department of Neurology, University of Michigan, Ann Arbor, Michigan, USA.

## Abstract

RNA-binding proteins (RBPs) are essential factors required for the physiological function of neurons, muscle, and other tissue types. In keeping with this, a growing body of genetic, clinical, and pathological evidence indicates that RBP dysfunction and/or gene mutation leads to neurodegeneration and myopathy. Here, we summarize the current understanding of matrin 3 (MATR3), a poorly understood RBP implicated not only in ALS and frontotemporal dementia but also in distal myopathy. We begin by reviewing MATR3’s functions, its regulation, and how it may be involved in both sporadic and familial neuromuscular disease. We also discuss insights gleaned from cellular and animal models of MATR3 pathogenesis, the links between MATR3 and other disease-associated RBPs, and the mechanisms underlying RBP-mediated disorders.

## Introduction

ALS is a neurodegenerative disease characterized by the loss of upper and lower motor neurons, resulting in weakness and paralysis. In contrast, frontotemporal dementia (FTD) involves behavioral and speech changes due to degeneration of neurons in the frontal and temporal lobes (1). Despite affecting disparate parts of the nervous system and manifesting in different symptoms, ALS and FTD share several clinical, genetic, and pathological features. In particular, RNA-binding proteins (RBPs) are integral players in both ALS and FTD pathogenesis, with mutations in a number of RBP-encoding genes causing familial ALS, FTD, or both. Even in individuals with sporadic disease with no known underlying mutation or family history — accounting for the majority of ALS and FTD — RBP mislocalization in affected nervous system regions is a signature pathological event (2–4). For many of these genes, the spectrum of affected tissues extends beyond neurons to skeletal muscle, manifesting as myopathies. One such gene encodes the highly conserved nuclear protein matrin 3 (MATR3), which possesses both DNA- and RNA-binding capacity. *MATR3* mutations were originally associated with inherited vocal cord and pharyngeal distal myopathy (VCPDM) and later recognized in patients with familial ALS and FTD. This Review will discuss basic MATR3 biology and its functions in nucleic acid processing in the nervous system, clinical evidence tying MATR3 to neuromuscular disease, and insights into MATR3-mediated pathogenesis from model systems and human postmortem tissue.

## Molecular and cellular biology of MATR3

### MATR3 function

#### MATR3 as a DNA-binding protein.

**Figure 1:**
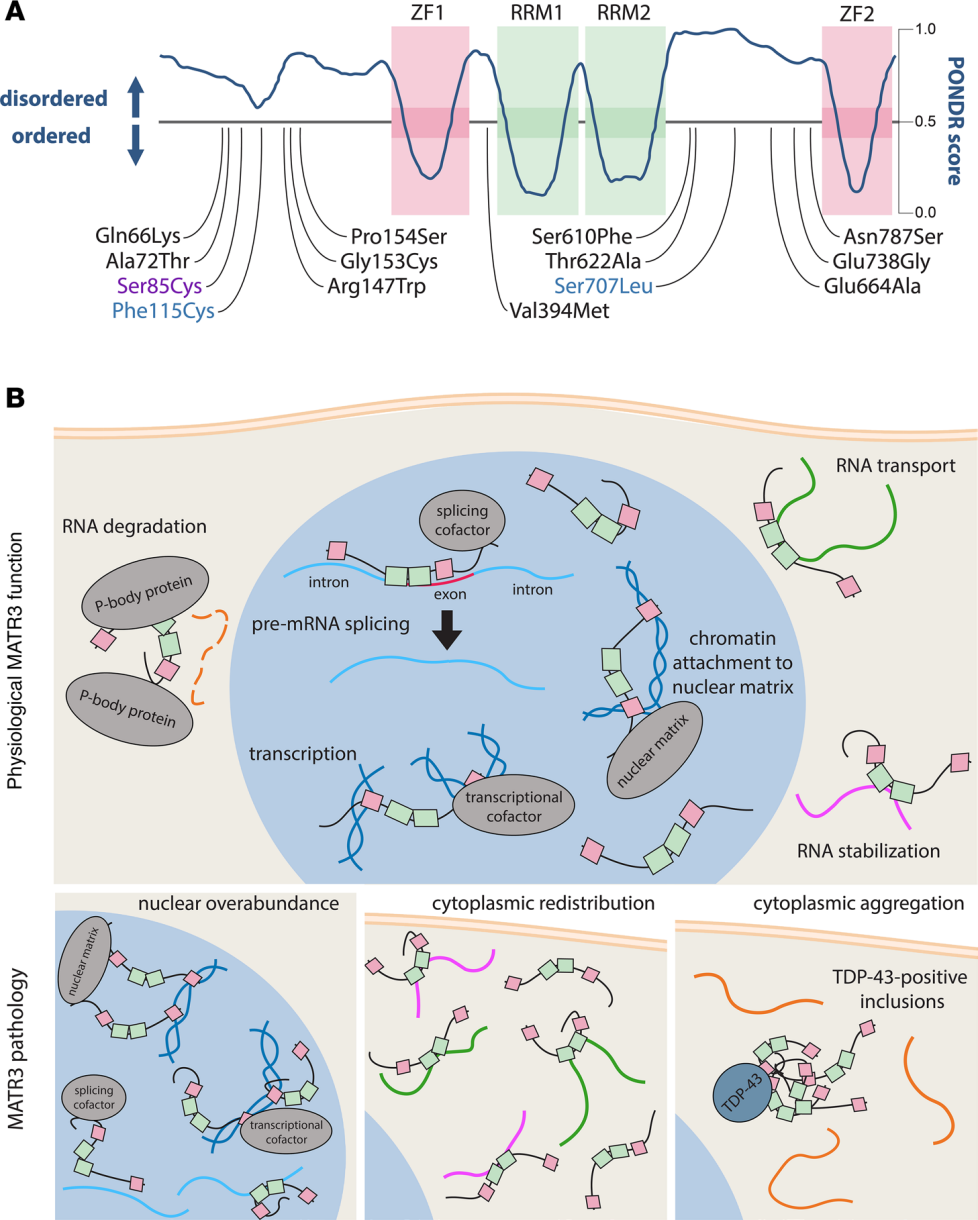
MATR3 domain structure and functions in normal and pathological contexts. (**A**) MATR3 has two zinc finger (ZF) and two RNA-recognition motif (RRM) domains, with the remainder of the protein consisting of an intrinsically disordered sequence as measured by a high Predictor of Natural Disordered Regions (PONDR) score (128). Pathogenic mutations are located across the disordered stretches of MATR3; although the majority of mutations reported to date are linked to ALS, a subset is implicated in ALS/distal myopathy (violet) or ALS/dementia (blue). (**B**) Although predominantly localized in the nucleus, MATR3 is tied to several nucleic acid–related processes in both nuclear and cytoplasmic compartments. Three distinct but not mutually exclusive patterns of MATR3 pathology are observed in neuromuscular disease: nuclear enrichment, cytoplasmic redistribution, and cytoplasmic aggregation. Nuclear overabundance is predicted to drive chromatin, transcriptional, and splicing aberrations. In addition, MATR3 redistribution and aggregation in the cytoplasm — representing cytosolic gain and loss of function, respectively — may disrupt RNA stability and transport.

MATR3 was initially identified as a major component of the nuclear matrix, the proteinaceous network responsible for organizing and maintaining nuclear architecture (5, 6). Consistent with this, a nuclear protein eventually recognized as MATR3 (7) was found in association with repetitive DNA sequences (8, 9). MATR3 possesses not only zinc finger (ZF) domains but also tandem RNA recognition motifs (RRMs), and the remainder of the protein consists of two large intrinsically disordered regions (IDRs), with the C-terminal IDR being highly acidic (Figure 1).

MATR3’s two ZF domains are of the C2H2 variety, which bind DNA but can also recognize RNA and mediate protein-protein interactions (10, 11). MATR3’s DNA substrates and the functional consequences of its DNA binding, however, remain poorly understood. Early work demonstrated that MATR3 recognizes repetitive, adenine/thymine-rich fragments isolated from rat liver DNA and that the recognition of these sequences is ZF dependent, as deletion of individual ZFs reduces binding measured by EMSAs. Deletion of both ZFs abolishes binding completely (12), suggesting that these domains function in an additive or cooperative fashion to bind DNA. Adenine/thymine-rich DNA sequences make up canonical scaffold/matrix attachment regions (S/MARs) (13, 14), sections of chromosomal DNA that serve as contact points between chromatin and the nuclear matrix. Protein components of the nuclear matrix can modulate gene expression by binding to S/MARs adjacent to genes or regulatory elements, thereby changing their accessibility to transcriptional and replication machinery (15–19). Imaging-based approaches have detected MATR3 in close proximity to functional genomic areas, such as transcriptional start and DNA replication sites (20, 21), supporting a possible role in tuning DNA availability.

Even so, the specific consequences of MATR3 DNA binding for gene expression are less clear. Using a luciferase reporter fused to a MATR3 DNA target, Hibino and colleagues demonstrated that luciferase activity is progressively inhibited by DNA methylation, which blocks MATR3 binding to the reporter, suggesting a potential role for MATR3 in transcriptional regulation (22). MATR3 phosphorylation greatly enhances its DNA-binding ability, indicating a physiological mechanism for regulating MATR3’s interaction with DNA (7). However, reporter methylation in these studies may have affected the binding of other transcription factors independent of MATR3, and it is unclear whether the changes in reporter expression were truly mediated by MATR3.

Additional evidence supports a potential function for MATR3 in cell type–specific gene regulation. Using ChIP-Seq in a rat pituitary cell line, Skowronska-Krawczyk et al. found that MATR3 binding is concentrated in noncoding areas of the genome and significantly overlaps with enhancer signals (23). The association of MATR3 with many of these sequences, as well as gene expression from these loci, appears to be indirect and is instead dependent on the pituitary-specific transcription factor Pit1. Similarly, MATR3 was identified as a DNA-binding protein capable of recognizing chromatin insulator sequences, but this interaction is also indirect and dependent on the transcription factor CTCF (24). Therefore, although MATR3 is capable of binding to chromatin in vitro via its ZF domains, it is currently unclear how or if MATR3 regulates gene expression or chromatin accessibility by direct association with DNA. More likely, MATR3 affects gene expression indirectly, by acting in concert with tissue- and cell-specific transcription factors.

In addition to transcriptional control, MATR3 functions in DNA repair, as indicated by the abnormal accumulation and impaired disassembly of nonhomologous end-joining repair factors at sites of DNA damage upon MATR3 knockdown in U2OS cells (25). MATR3 also binds to and stabilizes mRNA encoding Rad51, a key factor involved in homologous recombination, resulting in increased Rad51 levels and function (26). Under different circumstances, however, dsDNA damage triggers the formation of a complex including MATR3, p53, and long noncoding RNA that drives the expression of genes responsible for cell cycle arrest and modulation of apoptosis (27).

## MATR3 as an RBP

MATR3 has two tandem RRMs capable of binding RNA sequences in vitro and in vivo, and a number of studies have offered insights into the contributions of MATR3 to RNA metabolism. The first evidence of RNA binding came from Hibino et al., who identified an albumin-encoding mRNA sequence bound by MATR3 and, via EMSAs, showed that deletion of RRM1 and RRM2 impairs recognition by MATR3 (12). Since then, accumulating data have linked MATR3 to multiple points in the RNA life cycle. In U2OS cells, a C-terminally truncated MATR3 isoform localizes to cytoplasmic processing bodies (P-bodies), where it presumably functions as part of the RNA-induced silencing complex to degrade targeted RNAs (28). Consistent with this notion, proteomic analyses previously identified MATR3 as a component of argonaute-rich RNA-silencing complexes (29).

In other contexts, RNA IP followed by sequencing uncovered several MATR3 substrate RNAs that were stabilized by MATR3 binding, in agreement with a large-scale study of RBPs suggesting that MATR3 binding increases luciferase reporter levels (30, 31). MATR3 variants lacking RRM1 pull down many of the same RNA-dependent protein targets as full-length MATR3, whereas deletion of RRM2 prevents this, indicating that — as with other tandem RRM-harboring RBPs — one RRM may dominate in RNA binding (32–34). Furthermore, ZF domain deletion enhances MATR3 splicing activity, implying that MATR3’s DNA- and RNA-binding functions may compete and/or interfere with one another (35). Supporting this, ZF1 deletion promotes interaction of MATR3 with miR138-5p, and RRM removal interrupts this association (36). These data suggest that MATR3’s DNA-binding activity may antagonize its functions in RNA splicing and metabolism. In argument against a clean functional division between ZF and RRM domains, however, deletion of ZF2 reduces MATR3 association with miR138-5p, indicating potential overlap in nucleic acid binding between the two types of domains.

A large-scale in vitro study of RBP motifs determined that MATR3 recognizes a consensus AUCUU sequence in substrate RNA (37). This result was subsequently corroborated in human neuroblastoma (SH-SY5Y) cells using photoactivatable ribonucleoside-enhanced CLIP (PAR-CLIP). In these investigations, the majority of MATR3 sites were located in introns, and *MATR3* knockdown resulted in significant changes in exon cassette usage, suggesting that MATR3 functions as a splicing factor (38). Approximately equal numbers of novel exon skipping and inclusion events were noted upon MATR3 knockdown, but exons adjacent to introns with MATR3 binding sites were disproportionately included in the absence of MATR3. These observations suggest that MATR3 normally functions as an intronic splicing suppressor. In support of this conclusion, MATR3 represses exon inclusion in approximately two-thirds of differentially spliced genes in HeLa cells, implying that MATR3 represses splicing of most but not all of its substrate pre-mRNAs (35, 39). As with the DNA regulatory functions of MATR3, however, it remains unclear whether MATR3 directly or indirectly contributes to RNA splicing. For example, MATR3 and polypyrimidine tract binding protein 1 (PTBP1) share many substrates, physically interact with one another via the PTB RRM2 interacting (PRI) motif in MATR3, and act cooperatively to repress retrotransposons (40, 41). In addition to PTBP1, a number of protein-protein interaction screens and proteomics experiments have identified MATR3 in association with many other distinct RBPs and ribonuclear complexes, suggesting cooperative functions and broader roles in RNA metabolism beyond those uncovered to date (20, 42–50).

As with other RBPs, MATR3 demonstrates prominent self-association that is antagonized by RNA binding. Deletion of RRM2 — either in isolation or in combination with RRM1 — results in spherical droplet formation through liquid-liquid phase separation (LLPS). This phenomenon likely arises because of uninhibited interaction between MATR3’s IDRs, particularly the N-terminal IDR (51–53). Importantly, RNA binding–deficient TDP-43 and FUS variants also undergo LLPS (54–56). These data suggest that RBPs demix and form liquid-like droplets under low RNA conditions or when RNA binding is impaired via unchecked interactions between IDRs.

Somewhat surprisingly in light of its DNA- and RNA-binding capacity, MATR3 overexpression or knockdown results in relatively few detectable changes in gene expression. MATR3-YFP overexpression affects only a handful of genes in H4 neuroglioma cells compared with YFP alone (52), and *MATR3* knockdown in SH-SY5Y and HeLa cells results in expression changes for only a few dozen genes, despite affecting hundreds of alternative splicing events (35, 38). Nevertheless, the true magnitude or consequences of MATR3 loss may be cell type dependent. In myoblasts, for instance, MATR3 binds to and regulates several genes important for muscle differentiation and maturity, and MATR3 knockdown impairs the differentiation of these cells into mature myotubules (57). Interestingly, MATR3 exhibits dynamic changes in subcellular distribution as myoblasts mature into myotubes — although it is localized diffusely in the myoblast nucleoplasm, MATR3 rims the inner face of the nuclear envelope in myotubes, suggesting specialized roles for MATR3 in muscle development (58). One possibility is that this change reflects a shift in the function of MATR3 at each stage of differentiation, from DNA-mediated transcriptional regulation to RNA-mediated splicing activity or vice versa. Additional work is needed, however, to determine whether changes in MATR3 localization predict its function at the DNA or RNA levels, and whether MATR3 may serve similar roles in other cell types.

## MATR3 regulation

### MATR3 transcript variants.

Immature *MATR3* pre-mRNA undergoes alternative splicing, generating several unique transcripts that encode at least three district protein-coding variants. To date, only the full-length, 847 aa isoform has been studied. The two other species, 509 aa and 559 aa, lack the N-terminal IDR and ZF1. Rajgor et al. proposed the existence of a novel, C-terminally truncated isoform in U2OS cells, though this was not detectable by 3′ rapid amplification of cDNA ends (RACE) (28). The regulatory mechanisms that coordinate alternative *MATR3* splicing are unknown, as are the potential functions of N-terminally or C-terminally truncated MATR3 variants.

The *MATR3* transcript is also alternatively polyadenylated in a tissue-specific manner. Sequencing data demonstrate two different polyA signals within the *MATR3* 3′ UTR. The proximal polyA site is overrepresented in adult human cardiac and skeletal muscle cells (59), whereas all other cell types, including neurons and lymphoblasts, use the distal site almost exclusively. Unexpectedly, lymphoblasts from a patient with a balanced translocation interrupting the distal polyA site displayed a massive upregulation of the proximal polyA species and MATR3 protein levels. These results suggest that the proximal polyA signal may increase the stability and/or efficiency of *MATR3* mRNA translation. Abnormal intron retention, as observed in hepatocellular carcinoma, also results in increased *MATR3* mRNA abundance, further supporting the effects of alternative splicing on *MATR3* transcript stability (60) while implicating additional, as yet unknown mechanisms in MATR3 regulation at the RNA level.

### MATR3 abundance.

MATR3 levels are tightly regulated during development and in a cell type–specific fashion. In mice, MATR3 protein expression appears to be highest during fetal development but declines and ultimately stabilizes after birth (61). MATR3 levels vary considerably across cell types and are lowest in skeletal muscle and in the nervous system; such cell type–specific regulation may be particularly pertinent given that muscle and neurons are the two tissues most affected by MATR3-mediated disease.

On a more granular level, MATR3 expression within the brain varies considerably among individual neurons, as adjacent neurons of the same type located in the same brain region display varying MATR3 immunostaining intensities. A possible explanation for this heterogeneity may lie in the activity-dependent regulation of MATR3 abundance. In cerebellar neurons, NMDA receptor activation results in PKA-mediated MATR3 phosphorylation and subsequent degradation (62). Additionally, MATR3 is tightly bound by activated calmodulin, a major factor responsible for calcium-mediated signal transduction in response to neuronal depolarization (63). Differences in NMDA receptor activity between neurons may therefore account for the varying intensities of MATR3 staining observed in brain sections. Furthermore, given that expression of NMDA receptor subunits is developmentally regulated (64), an analogous mechanism may explain differences in MATR3 expression over development.

### MATR3 localization and posttranslational modifications.

Subcellular MATR3 distribution and abundance are regulated by several factors, including primary sequence, functional domains, and posttranslational modifications (PTMs). Nuclear localization signals (NLSs) effectively concentrate MATR3 within the nucleus but appear to be differentially utilized by distinct cell types. In chicken lymphoma cells, for example, both arms of a bipartite NLS (65) are necessary for targeting MATR3 to the nucleus, while in rat Ac2F cells (12), a separate stretch of positively charged aa in the middle of the C-terminal IDR drives MATR3 nuclear localization. In primary rat cortical neurons, however, deletion of this latter NLS has no effect; rather, the N-terminal arm of the bipartite NLS is both necessary and sufficient for MATR3 nuclear localization (51). In NLS mutant–expressing neurons, cytoplasmic MATR3 forms discrete granules that undergo transport along neuronal processes. Given the propensity for MATR3 to form liquid droplets in the absence of functional RNA binding, and the relatively low RNA concentrations in the cytoplasm compared with the nucleus, we suspect that these structures represent phase-separated MATR3 droplets (55, 66).

PTMs may also regulate MATR3 nucleocytoplasmic shuttling and function. Alpha herpesvirus infection does not change host MATR3 localization in human fibroblasts, but transduction with viral variants lacking a homologous serine/threonine kinase results in a striking redistribution of MATR3 to the cytoplasm (67). These results suggest that phosphorylation of MATR3 or another target upon infection with WT viruses maintains nuclear MATR3 localization. In agreement, application of a broad-spectrum kinase inhibitor resulted in the accumulation of cytoplasmic MATR3 in NIH3T3 cells, indicating an endogenous phosphorylation pathway in mammalian cells responsible for nuclear MATR3 enrichment (65). Additional investigations suggested that MATR3 phosphorylation at Ser208 promotes nuclear localization, as expression of the phosphorylation-null Ser208Ala variant in fibroblasts leads to MATR3 nuclear clearance (68). Of note, ATM phosphorylates Ser208 in response to DNA damage, an event that is necessary for MATR3 function in at least certain arms of the DNA damage response (25).

PTMs also affect the ability of MATR3 to recognize nucleic acids. Although the specific phosphoresidues are unknown, phosphorylation enhances MATR3 binding to adenine/thymine-rich DNA (7) as well as RNA sequences (69). These effects may be closely related to MATR3 subcellular localization, given that nuclear MATR3 is more heavily phosphorylated than cytoplasmic MATR3 (12). Where these PTMs are located and how they alter substrate recognition, however, remain unexplored.

## MATR3 in neuromuscular disease

### Spectrum of MATR3-mediated disease.

As described above, MATR3 was first implicated in human disease in an American family with an autosomal dominant form of distal myopathy with VCPDM (70). The causative gene was localized to chromosome 5q31, and subsequent investigations of a Bulgarian family with VCPDM revealed a Ser85Cys mutation in *MATR3* affecting a highly conserved aa within the N-terminal IDR that segregated with disease (71). Several additional families with MATR3(Ser85Cys)-linked VCPDM have since been described. These individuals are weak because of atrophy of distal limb muscles as well as proximal muscles of the pharynx and diaphragm (72). Microscopically, affected muscles exhibit atrophic fibers with rimmed vacuoles, internalized nuclei, and, at the end stage, fatty replacement (73–75). Immunostaining reveals cytoplasmic MATR3 in dystrophic muscle (76), in addition to myofiber inclusions rich in TDP-43, p62/SQSTM1, and ubiquitin (77, 78). Electromyography (EMG) demonstrates a myopathic pattern similar to related myopathies (79, 80) with variable degrees of neurogenic changes, consistent with a primarily myogenic form of disease (70, 73, 78). Biochemical analysis of MATR3 protein from patient samples shows no difference in abundance (76), but instead the accumulation of detergent-insoluble MATR3 that is more resistant to extraction (71).

Exome sequencing identified several novel *MATR3* mutations in patients with familial ALS or combined ALS/FTD. Four missense mutations, including Ser85Cys, located in MATR3’s IDRs were associated with ALS with or without cognitive deficits (81). Since then, a number of exome-sequencing studies have reported ALS-associated missense mutations concentrated within the disordered regions of the protein (82–86). The vast majority of these were identified in patients with ALS, though the Ser707Leu mutant was implicated in combined ALS/FTD. Moreover, two splice-site mutations were found in ALS patients, one in the 5′ UTR and the other predicted to add 24 new residues to the N-terminus of the 559 residue MATR3 isoform (84). In addition, one individual heterozygous for the Leu145Phe *SOD1* variant and a novel Arg841Cys *MATR3* mutation exhibited a pseudopolyneuritic form of ALS. Conclusions regarding the pathogenicity of this *MATR3* mutation are complicated by the presence of another ALS-causing gene variant, however (87).

Despite accumulating genetic evidence that testifies to the relationship between *MATR3* mutations and human disease, the consequences and tissue specificity of the Ser85Cys mutation remain controversial. This point mutation, first identified in families with VCPDM displaying myogenic and neurogenic EMG features (70, 71), was later associated with slowly progressive ALS and upper motor neuron involvement based on the presence of brisk reflexes in some patients (81). Similar neurogenic features have since been identified in a number of patients with the Ser85Cys mutation by antemortem (clinical examination, EMG) and postmortem (pathological) studies (73, 74, 78, 88). Taken together, these data suggest that the Ser85Cys mutation can cause myopathy or motor neuron disease, perhaps depending on unrecognized genetic or environmental modifiers. Further phenotyping in addition to detailed genetic analyses are needed to fully understand the spectrum of disease related to the MATR3(Ser85Cys) mutation.

The degree of dementia also appears to vary considerably in individuals with disease-associated *MATR3* mutations. The initial study linking *MATR3* mutations to ALS identified a Phe115Cys mutation in a patient with cognitive deficits, though this was reported only as dementia without detailed characterization (81). More recently, however, the Ser707Leu mutation was found in Italian patients displaying cognitive and behavioral symptoms consistent with FTD (85). To date, these two reports are the only evidence linking *MATR3* mutations to cognitive symptoms. Additional genetic studies coupled with detailed clinical descriptions are necessary to determine the extent to which different neuron subtypes and regions of the CNS are affected by MATR3-linked disease.

### MATR3 pathology.

In normal neurons, MATR3 adopts a granular nuclear localization. Individuals with familial ALS due to the highly prevalent *C9orf72* hexanucleotide repeat expansion show diffuse cytoplasmic MATR3 staining as well as cytoplasmic MATR3-positive inclusions in spinal motor neurons (81, 89). An identical pattern was observed in a patient with the MATR3(Phe115Cys) mutation (81), and cytoplasmic MATR3 aggregates are also found in patients with *FUS* mutation–linked ALS (89), suggesting a conserved pattern of MATR3 mislocalization in disease similar to that displayed by TDP-43. Importantly, these studies also highlighted MATR3 mislocalization in sporadic ALS (sALS), which accounts for more than 80% of incident ALS cases. Spinal cord samples from patients with sALS display strong nuclear MATR3 staining as well as cytoplasmic MATR3 localization, indicating abnormalities not only in MATR3 distribution but also abundance. Similarly, RNA sequencing of postmortem patient tissue indicates that *MATR3* expression increases in mild and moderate disease stages before dropping in late stages (90), supporting dysregulation of MATR3 expression as well as localization in ALS.

Subsequent investigations confirmed the presence of MATR3 pathology in spinal motor neurons of ALS patients but failed to replicate changes in MATR3 abundance with disease (91). In some motor neurons, MATR3 appears to be diffusely distributed in the cytoplasm, while in others MATR3 takes on a granular pattern or accumulates within large inclusions that costain for TDP-43. Although all MATR3-positive inclusions contain TDP-43, not all TDP-43–positive inclusions stain for MATR3, and TDP-43–positive inclusions are substantially more numerous than MATR3-positive deposits.

These observations suggest that, although both RBPs can undergo redistribution from the nucleus to the cytoplasm in disease, TDP-43 mislocalization may precede MATR3 pathology. Even so, the precise nature of the relationship between MATR3 and other ALS proteins requires further study, as does the discrepancy in reports of MATR3 overabundance in sALS cases.

MATR3 pathology can also be found in neurodegenerative conditions outside of ALS and FTD. Cytoplasmic MATR3 was recently detected in subiculum from patients with Alzheimer disease (AD) but not age-matched controls. Of note, these authors described not only increased MATR3 levels and diffusely cytoplasmic MATR3 immunostaining in affected neurons, but also ringed MATR3 deposits surrounding granulovacuolar degenerative bodies in AD neurons (92). In cultured cells, amyloid-β species drive MATR3 phosphorylation, but it is unknown whether this is the mechanism responsible for the MATR3 deposition noted in AD patients (93).

## Dysregulation in disease models

A number of investigators have attempted to recapitulate MATR3-mediated disease using both cellular and animal models. Pathogenic MATR3 mutants show no clear differences in nucleocytoplasmic localization or protein levels in comparison to MATR3(WT) in myoblasts or neurons (51, 94). Proteomic studies of MATR3 binding partners in a mouse spinal neuron/neuroblastoma hybrid cell line identified a distinct set of nuclear transport factors that differentially interact with pathogenic MATR3 variants. These factors, including components of the transcription and export (TREX) complex, were not confirmed in similar experiments conducted in HEK293T cells, suggesting that pathogenic MATR3 mutants may selectively associate with TREX complex members in neuronal cells (50, 52), thereby impairing nuclear mRNA export in a cell type–specific manner.

Among the disease-associated MATR3 mutations investigated in model systems, the Ser85Cys variant displays several unique properties. Unlike MATR3(WT), MATR3(Ser85Cys) was nearly absent from the soluble nuclear fraction of patient lymphoblasts but was instead concentrated in the insoluble fraction (71). Similarly, low levels of soluble MATR3(Ser85Cys) were detected in transfected HEK293T cells; although this was initially attributed to destabilization of the mutant protein (81), subsequent studies showed only minor changes in the turnover of MATR3(Ser85Cys) compared with MATR3(WT). Instead, MATR3(Ser85Cys) is markedly more resistant to detergent extraction and preferentially accumulates within the insoluble fraction of transfected cells (51). Even MATR3(WT) becomes markedly insoluble under conditions of thermal or proteotoxic stress, suggesting that the Ser85Cys mutation may lower the threshold for this behavior, rather than introducing novel properties per se (95, 96). Furthermore, in MATR3 variants that are unable to bind RNA and undergo LLPS, the Ser85Cys mutation dramatically affects the internal dynamics of MATR3 within liquid-like droplets. RNA binding–deficient MATR3(Ser85Cys) forms viscous hydrogel-like structures in primary neurons, and irregular fibrillar structures instead of spheres in C2C12 myoblasts (51, 52). Perhaps related to these biophysical phenotypes, the formation of liquid droplet-like cytoplasmic stress granules is impaired in fibroblasts from Ser85Cys patients compared with those from healthy controls, and Ser85Cys but not WT protein enhances the aggregation of cytoplasmically targeted TDP-43 when overexpressed (97). Whether and how these features explain the distinctive clinical picture of VCPDM associated selectively with the Ser85Cys mutation remains unknown.

Insights into MATR3-mediated disease have also emerged from in vivo modeling in *Drosophila*, which lack a MATR3 homologue. Two independent groups found that MATR3 expression in *Drosophila* results in shortened lifespan and motor deficits, with disease-associated mutants exhibiting increased toxicity over MATR3(WT) (98, 99). Notably, both studies found that Ser85Cys is uniquely insoluble in flies, recapitulating findings from cultured cells (51, 52). Robust wing defects caused by muscle-specific MATR3 expression were accentuated by pathogenic mutations, particularly Ser85Cys; this phenotype formed the basis for an RNAi screen that uncovered genes related to axonal transport as enhancers of toxicity, suggesting that alterations in intracellular trafficking may be involved in MATR3 pathogenesis. A separate RBP-targeted RNAi screen revealed that hnRNPM knockdown extends the lifespan of flies expressing Ser85Cys and Phe115Cys pathogenic mutants but not MATR3(WT). hnRNPM and MATR3 share many RNA substrates, and their respective binding sites are located in close proximity. It is therefore possible that MATR3 acts in concert with hnRNPM to mediate RNA dysfunction and toxicity in *Drosophila*.

Multiple groups have attempted to model MATR3-related disease in mice. Homozygous knockout of murine *Matr3* is perinatally lethal, indicating that MATR3 is necessary for viability (100). In a separate model, overexpression of human MATR3(WT) or MATR3(Phe115Cys) in skeletal muscle results in age-dependent muscle fiber degeneration with extensive vacuoles, internalized nuclei, and gross atrophy. Despite equivalent amounts of transgene mRNA, expression of MATR3(Phe115Cys) was greater than MATR3(WT), and only MATR3(Phe115Cys) animals demonstrated gross motor impairment, implying mutation-specific effects on translational control, turnover, or both (101). For unknown reasons, neither MATR3(Phe115Cys) nor MATR3(WT) was detectable within the spinal cord of these animals, despite use of the MoPrP promoter, which typically drives high CNS expression (102).

Similar myopathic changes were observed by intramuscular adeno-associated virus delivery of human MATR3(Ser85Cys) or MATR3(WT) in mice, including myofiber atrophy, internalized nuclei, and upregulation of muscle repair genes (103). Sarcoplasmic inclusions rich in MATR3 and p62/SQSTM1 were observed in transduced muscle sections, mirroring the pathology of inclusion body myopathy in humans (104). Despite similar expression of exogenous protein, this phenotype was more severe with MATR3(Ser85Cys) than MATR3(WT), supporting enhanced pathogenicity of mutant MATR3. Notably, analogous sarcoplasmic aggregates rich in MATR3 and p62/SQSTM1 are observed in muscle tissue from patients with VCPDM who carry the Ser85Cys mutation, lending clinical relevance to these findings (73, 77, 78). To investigate CNS-specific effects of MATR3 upregulation, Zhang and colleagues also generated transgenic mice expressing MATR3(Ser85Cys) under control of the CMV promoter. These animals develop age-dependent motor impairment with muscle degeneration, similar to that seen in adeno-associated virus–injected animals, but also demonstrate progressive spinal cord pathology with motor neuron loss, astrogliosis, microgliosis, and mislocalization of MATR3 and TDP-43. Although the striking CNS pathology is consistent with ALS, these observations are complicated by the unchanged levels of full-length MATR3 in transgenic mice compared with nontransgenic controls and the lack of MATR3(WT) transgenic animals for comparison.

A Ser85Cys mutation knockin model has also provided important pathogenic insights in the context of physiological MATR3 dosage (100). Homozygous knockin mice display age-dependent motor impairment, muscle denervation and pathology, and neuroinflammation. Strikingly, these animals also show marked cerebellar degeneration and loss of MATR3 within Purkinje neurons at end stage. Significant motor neuron loss was not noted, but approximately half of spinal α-motor neurons in homozygous knockin mice also exhibit diminished MATR3 immunoreactivity, with many others staining positive for intranuclear MATR3 inclusions. This model raises fascinating questions for future studies regarding the effects of the Ser85Cys mutation on MATR3 regulation in Purkinje and motor neurons and the relevance of cerebellar pathology for human MATR3-linked neurodegeneration.

## Similarity with other RBPs

MATR3 shares many similarities with other RBPs implicated in neuromuscular disease. MATR3 belongs to a subset of RBPs that are linked not only to the neurodegenerative disorders ALS and FTD but also to muscular disease. Although mutations in the genes encoding RBPs such as TDP-43 or FUS lead primarily to ALS, *MATR3* mutations can result in additional disorders such as FTD or VCPDM. Similar pleiotropy is also observed with *VCP*, *TIA1*, *hnRNPA1*, and *hnRNPA2B1* mutations, which can affect the CNS, skeletal muscle, and/or bone (105–107). Individual differences among genes and clinical phenotypes may be important for disease mechanisms in each case. For instance, *VCP*, *hnRNPA1,* and *hnRNPA2B1* mutations cause multisystem proteinopathy with Paget’s disease of bone, inclusion body myopathy, ALS, and FTD; *TIA1* and *MATR3* mutations result in distal myopathy in addition to ALS and FTD.

How mutations in these widely expressed proteins drive tissue-specific disease — and why their disease spectra differ — are currently not well understood. One promising explanation may lie in the dysregulation of pathways unique to certain cells. VCP degrades the NF-κB inhibitor IκB, thereby suppressing osteoclast activity and bone resorption (108, 109). Pathogenic *VCP* mutations promote IκB clearance, and VCP-mediated disease models show enhanced NF-κB signaling and osteoclast activation phenotypes, suggesting mutant VCP instigates bone pathology by disinhibiting NF-κB (110, 111). It is possible that similar cell type–specific functions for MATR3 and other RBPs dictate the range of phenotypes affecting muscle as well as neurons within the ventral spinal cord, motor cortex, and frontotemporal lobe.

All of these genes except *VCP* encode for IDR-containing RBPs that undergo phase separation as part of their normal functions in RNA splicing, degradation, sequestration, and transport. Notably, the physiological LLPS of RBPs is dynamic and reversible, with factors such as substrate binding and PTMs tuning self-assembly (55, 112–118). Disease-associated mutations may interrupt physiological LLPS regulation, promoting aberrant liquid-to-solid phase transitions that eventually lead to irreversible RBP aggregates characteristic of ALS, FTD, and inclusion body myopathy (106, 107, 119–123).

Upon RRM2 deletion, MATR3 rapidly undergoes LLPS, consistent with a model in which RNA binding pulls RBPs apart from each other. The extent to which MATR3 phase separation is necessary for its functions in nucleic acid processing remains unknown, as are the physiological factors regulating this process. Moreover, data from multiple groups suggest that the Ser85Cys mutant dramatically affects the biophysical properties of MATR3 liquid droplets, analogous to what has been reported for pathogenic mutations in other disease-linked RBPs (51, 52, 71, 103). It is currently unclear whether other MATR3 mutations have similar effects on pathological phase transitions or whether they drive disease through alternative mechanisms.

## Conclusions and future directions

Mutations in the MATR3-encoding gene are responsible for neuromuscular disease, implying that this protein is critical for maintaining neurons as well as muscle, but how *MATR3* mutations affect its function and/or contribute to disease pathogenesis remains unclear. Potentially because of the similarity between MATR3 and other neurodegenerative disease–associated RBPs, the majority of investigations to date have focused on MATR3’s influence on pre-mRNA splicing and RNA regulation. In contrast, much less in known about the consequences of MATR3 DNA binding and whether the dysregulation of MATR3 chromatin targets is involved in disease. Indeed, it is currently unclear whether MATR3 binds chromatin directly in vivo or instead requires tissue-specific factors for DNA association.

Another outstanding question concerns the phenotypic spectrum of MATR3-linked disease and how *MATR3* mutations can cause motor neuron degeneration, cortical neuron loss, and/or myopathy. The Ser85Cys mutation is capable of driving both myogenic and neurogenic disease, suggesting the presence of specific environmental or genetic factors that modify tissue specificity. Moving forward, the continued identification and thorough clinical testing of patients with *MATR3* mutations will be essential if we are to uncover the identity and function of these modifiers. At present, many identified *MATR3* variants are singletons; therefore, it will be critical to not only verify these mutations in other patients and families, but also confirm their pathogenicity and investigate mutation-specific pathways in model systems. Of note, the Phe115Cys MATR3 variant was recently reclassified as likely nonpathogenic based on its incomplete segregation with disease and the identification of a mutation in another ALS-linked gene, *KIF5A*, segregating with ALS/FTD in the original kindred (124). This result underscores the vital need to reexamine variant pathogenicity in light of new data.

Cellular and animal models have been pivotal for our understanding of MATR3-linked neuromuscular disease, and we expect new models to offer further insights. Induced pluripotent stem cell (iPSC) technology represents a particularly promising approach for understanding MATR3 function, and one patient-derived iPSC line has already been created (125). iPSCs would be particularly useful in studying tissue-specific functions because these cells can be differentiated into isogenic motor neurons, cortical neurons, and skeletal muscle cells. In addition to enabling the identification of cell type–specific MATR3 functions and pathogenic pathways triggered by mutant *MATR3* variants, iPSCs may facilitate the identification of elements of MATR3 biology that are unique to human neurons, as has recently been the case for TDP-43 (126–127).

Despite the identification of MATR3 as a nuclear matrix component nearly three decades ago, important questions remain about its diverse functions in nucleic acid processing as well as its involvement in sporadic and inherited neuromuscular disorders. It is our expectation that continued research in this exciting field will uncover key elements of MATR3 physiology and pathophysiology, laying the groundwork for the rational development of effective therapies for MATR3-mediated disease.

## Version 1 01/11/2021

Electronic publication
